# The complete chloroplast genome of *Prunus takasagomontana*, a deciduous flowering cherry native to Taiwan island

**DOI:** 10.1080/23802359.2022.2102946

**Published:** 2022-07-29

**Authors:** Dongyue Jiang, Yingang Li, Lifang Zhang, Xin Shen

**Affiliations:** Zhejiang Academy of Forestry, Hangzhou, PR China

**Keywords:** *Prunus takasagomontana*, chloroplast genome, pair-end sequencing, phylogenetic analysis

## Abstract

*Prunus takasagomontana* Sasaki 1931 is a deciduous flowering cherry endemic to Taiwan island, China. Here, we first report the complete chloroplast genome of *P. takasagomontana*. The complete chloroplast genome of *P. takasagomontana* is 157,946 bp in length, which is comprised of a pair of inverted repeat (IR) regions of 26,437 bp, a small single-copy (SSC) region of 19,145 bp, and a large single-copy (LSC) region of 85,927 bp. A total of 129 genes are annotated, including 84 protein-coding genes, 37 tRNA genes, and eight rRNA ribosomal genes. The phylogenetic analysis showed that *P. takasagomontana* is sister to *P. serrulata* var. *spontanea*.

*Prunus takasagomontana* Sasaki 1931 (Rosaceae) is a wild flowering cherry endemic to Taiwan island, China (Huang 1993). Since *P. takasagomontana* is distributed in Lala Shan, north of Taiwan island, it is also called Lala flowering cherry. Compared with other wild flowering cherry species native to Taiwan island on characteristics of the flower, *P. takasagomontana* has a bell-shaped calyx tube and white flower. This species is a valuable ornamental plant, which can be used as garden trees, street trees, flowers, and potted plants. However, the phylogenetic relationships of *P. takasagomontana* with other *Cerasus* species remain unclear due to the similar morphological features and a lack of phylogenetic signal (Huang et al. 2006). Here, we reported the chloroplast genome of *P. takasagomontana* which will benefit future studies on the conservation and phylogeny of wild flowering cherry on Taiwan island.

The fresh leaves of *P. takasagomontana* were collected from Lala Shan, Taiwan, China (24°42′35″N 121°25′57″E). A voucher specimen (no. JDYY039) was deposited in the Herbarium of Zhejiang Academy of Forestry, Hangzhou, China (Xin Shen, shenxinjdy@126.com). The genomic DNA was extracted from leaf samples using Hi-DNAsecure Plant Kit (Tiangen, Beijing, China), and used to construct a short-insert (<800 bp) paired-end sequencing library. Paired-end sequencing was performed on the HiSeq X Ten analyzer (Illumina, San Diego, CA) at Novogene Co. Ltd. (Beijing, China). The chloroplast DNA sequences were manually adjusted using Geneious 2022.0.1 (Kearse et al. 2012) with *P. takesimensis* (MG754959) (Cho et al. 2018) as a reference, and assembled by SPAdes (Bankevich et al. 2012). The chloroplast genome annotation and correction were conducted using Plann and Sequin (Huang and Cronk 2015).

The size of the chloroplast genome of *P. takasagomontana* is 157,946 bp in length with 36.7% GC content, which contains a pair of inverted repeat (IR, 26,437 bp) regions, a small single-copy (SSC, 19,145 bp) region, and a large single-copy (LSC, 85,927 bp) region. The chloroplast genome encoded a total of 129 genes, containing 84 protein-coding genes (PCGs), 37 tRNA genes, and eight rRNA ribosomal genes. Among these genes, 112 genes are single copy, while six PCGs, seven tRNA genes, and four rRNA genes in IR regions are duplicated.

The complete chloroplast genome sequences of other 19 *Prunus* species were used to construct the phylogenetic tree to clarify the phylogenetic position of *P. takasagomontana* with two *Malus* species and one *Spirea* species as the outgroup. The maximum-likelihood (ML) was performed using PhyloSuite with 1000 bootstrap replicates (Zhang et al. 2020). The best-fit model was TVM + F+I according to the BIC criterion using ModelFinder (Kalyaanamoorthy et al. 2017). The phylogenetic analysis demonstrated that *P. takasagomontana* was sister to *P. serrulata* var. *spontanea*, and had a close relationship with *P. takesimensis* and *P. maximowiczii* (Figure 1).

**Figure 1.**
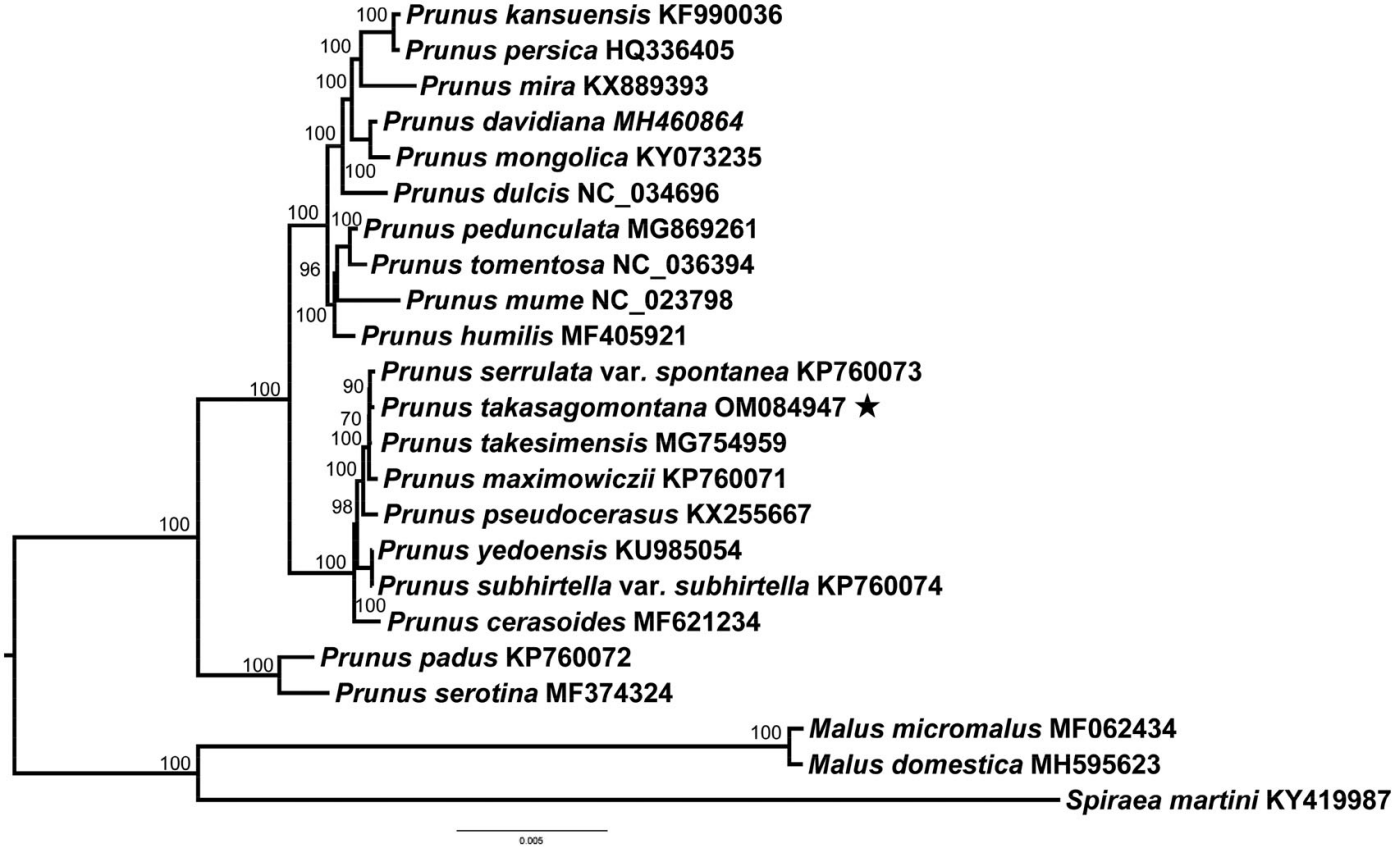
Maximum-likelihood phylogenetic tree for *Prunus takasagomontana* based on other 22 complete chloroplast genomes with 1000 bootstrap replicates.

## Author contributions

X. S. and DY. J. contributed to the conception. DY. J., X. S., and YG. L. designed the work and collected the plant materials. LF. Z., YG. L., and DY. J. performed experiments. DY. J., LF. Z., and YG. L. analyzed and interpreted the data. X. S., DY. J., and LF. Z. wrote the manuscript. All authors were involved in the final approval of the version to be published. All authors agree to be accountable for all aspects of the work.

## Ethical approval

Collection and study of plant material were conducted according to the guidelines provided by the Zhejiang Academy of Forestry. Permission was granted by the National Natural Science Foundation of China.

## Data Availability

The data that support the findings of this study are openly available in GenBank of NCBI at https://www.ncbi.nlm.nih.gov/ under the accession no. OM084947. The associated BioProject, SRA, and Bio-Sample numbers are PRJNA795269, SRR17594920, and SAMN24699336, respectively.
